# *In vitro *activity of tigecycline in combination with various antimicrobials against multidrug resistant *Acinetobacter baumannii*

**DOI:** 10.1186/1476-0711-8-18

**Published:** 2009-05-21

**Authors:** Luigi Principe, Silvia D'Arezzo, Alessandro Capone, Nicola Petrosillo, Paolo Visca

**Affiliations:** 1 National Institute for Infectious Diseases "Lazzaro Spallanzani", Via Portuense 292, 00149 Rome, Italy; 2 Department of Biology, University Roma Tre, Viale Marconi 446, 00146 Rome, Italy

## Abstract

**Background:**

Infections sustained by multidrug-resistant (MDR) and pan-resistant *Acinetobacter baumannii *have become a challenging problem in Intensive Care Units. Tigecycline provided new hope for the treatment of MDR *A. baumannii *infections, but isolates showing reduced susceptibility have emerged in many countries, further limiting the therapeutic options. Empirical combination therapy has become a common practice to treat patients infected with MDR *A. baumannii*, in spite of the limited microbiological and clinical evidence supporting its efficacy. Here, the *in vitro *interaction of tigecycline with seven commonly used anti-*Acinetobacter *drugs has been assessed.

**Methods:**

Twenty-two MDR *A. baumannii *isolates from Intensive Care Unit (ICU) patients and two reference strains for the European clonal lineages I and II (including 3, 15 and 6 isolates that were resistant, intermediate and susceptible to tigecycline, respectively) were tested. Antimicrobial agents were: tigecycline, levofloxacin, piperacillin-tazobactam, amikacin, imipenem, rifampicin, ampicillin-sulbactam, and colistin. MICs were determined by the broth microdilution method. Antibiotic interactions were determined by chequerboard and time-kill assays. Only antibiotic combinations showing synergism or antagonism in both chequerboard and time-kill assays were accepted as authentic synergistic or antagonistic interactions, respectively.

**Results:**

Considering all antimicrobials in combination with tigecycline, chequerboard analysis showed 5.9% synergy, 85.7% indifference, and 8.3% antagonism. Tigecycline showed synergism with levofloxacin (4 strains; 16.6%), amikacin (2 strains; 8.3%), imipenem (2 strains; 8.3%) and colistin (2 strains; 8.3%). Antagonism was observed for the tigecycline/piperacillin-tazobactam combination (8 strains; 33.3%). Synergism was detected only among tigecycline non-susceptible strains. Time-kill assays confirmed the synergistic interaction between tigecycline and levofloxacin, amikacin, imipenem and colistin for 5 of 7 selected isolates. No antagonism was confirmed by time-kill assays.

**Conclusion:**

This study demonstrates the *in vitro *synergistic activity of tigecycline in combination with colistin, levofloxacin, amikacin and imipenem against five tigecycline non-susceptible *A. baumannii *strains, opening the way to a more rationale clinical assessment of novel combination therapies to combat infections caused by MDR and pan-resistant *A. baumannii*.

## Background

*Acinetobacter baumannii *has emerged as a leading nosocomial pathogen, particularly in Intensive Care Units (ICUs), where several outbreaks have been described [1]. The epidemic potential and the clinical severity of *A. baumannii *infections are primarily related to the propensity of this organism to develop resistance to a variety of antimicrobial agents, including broad-spectrum beta-lactams, aminoglycosides, fluoroquinolones and carbapenems [2].

Carbapenems remain drugs of choice for the treatment of *A. baumannii *infection, but their efficacy can be compromised by the spread of novel class D carbapenemases [3,4]. As a result, carbapenem-intermediate or -resistant *A. baumannii *isolates are becoming increasingly prevalent in several countries [5]. Colistin, an old antibiotic from the polymixin group, is very effective against multidrug-resistant (MDR) *A. baumannii *isolates, but the emergence of resistance has occasionally been experienced [6]. Moreover, unfavourable pharmacokinetic properties and possible adverse effects restrict its clinical use [7,8]. Tigecycline, a new semi-synthetic tetracycline, has provided hope for the treatment of *A. baumannii *infections, including carbapenem-resistant isolates [9,10]. However, *A. baumannii *isolates showing reduced susceptibility to tigecycline have recently been identified in Israel [11], Spain [12], Italy [13], USA [14], and China [15]. Moreover, the exposure to sub-MIC tigecycline concentrations has been shown to facilitate the development of resistance in *A. baumannii *both *in vitro *and *in vivo *[16-18]. In this scenario, combination therapy has become the ultimate resource to treat MDR and pan-resistant *A. baumannii *infections [19], but its actual efficacy is unclear from a microbiological and clinical viewpoint. Previous investigations revealed an overall indifferent effect of tigecycline in combination with other antimicrobial agents commonly used against *Acinetobacter *spp., including carbapenems, fluoroquinolones, rifampicin, ampicillin-sulbactam, piperacillin-tazobactam, polymyxin B and colistin [20-22]. Only few clinical studies reported successful *in vivo *treatment of MDR *A. baumannii *infection with tigecycline in combination with colistin, meropenem, piperacillin-tazobactam and cotrimoxazole [23,24]. However, these results were not supported by an *in vitro *synergy study. Recently, tigecycline/amikacin synergistic interactions have been observed *in vitro *[22,25], but rarely in case of carbapenem-resistant *A. baumannii *strains [25].

The aim of this study was to investigate the *in vitro *activity of tigecycline in combination with a variety of commonly used antimicrobial agents against MDR *A. baumannii *isolates, including tigecycline intermediate and resistant strains. Part of this work has been presented at the 48th ICAAC meeting, Washington DC (USA), 2008 (Poster C1-3817).

## Methods

### Bacterial isolates and epidemiological typing

Twenty-two *A. baumannii *isolates were selected from a well-characterized set of MDR *A. baumannii *isolates collected in the period January 2004–June 2005 from the ICU of 7 general hospitals of the Rome (Italy) urban area [13,26]. Twenty *A. baumannii *isolates were obtained from clinical specimens of ICU patients, and 2 were recovered from the ICU environment (Table 1). None of the patients underwent previous treatment with tigecycline. The collection also comprises an index strain (study code 115, also called ACICU) from an ICU outbreak [27], whose complete genome has recently been sequenced [28]. The two prototypic strains for the epidemic European clonal lineages I (RUH875) and II (RUH134) were included as reference [29]. The selection criteria for *A. baumannii *isolates were based on hospital of origin (representing 7 hospitals, named A to G, in the Rome urban area), tigecycline susceptibility (6 sensitive; 15 intermediate; 3 resistant), molecular type (RAPD fingerprint, pulsotype and sequence group as described) [26] and antibiotic resistance profile (Table 1). Since there is no widely accepted definition for MDR *A. baumannii*, [30], hereafter we shall refer to the MDR phenotype as diminished susceptibility to ≥ 2 of the following drug classes: antipseudomonal cephalosporins, antipseudomonal carbapenems, β-lactam-β-lactamase inhibitor combinations, antipseudomonal fluoroquinolones and aminoglycosides, according to ref. [31]. Twenty-one isolates were genetically related to either the European clonal lineage I (6 isolates) or II (15 isolates), and indicated as type 2 or 1, respectively, upon sequence group analysis, RAPD, and pulsotyping. Typing data have been published elsewhere [26]. In addition, 3 *A. baumannii *isolates (study codes 50, 75 and 105) showing a variant molecular type were included. Isolates belonging to RAPD types 1, 1a and 4 were resistant to carbapenems, while those belonging to RAPD types 2 and 2a were susceptible (Table 1).

**Table 1:** Characteristics of *A. baumannii *isolates^a^

Study code (Hospital)	Source (isolation date)	Sequence group	RAPD type	Pulsotype	Antibiotic resistance profile ^b^
5 (A)	Respiratory secretions (06/15/04)	1	1	1	LVX TZP AMK IPM RIF SAM TIG
11 (B)	Respiratory secretions (05/15/04)	1	1	1	LVX TZP AMK IPM RIF TIG
16 (B)	Respiratory secretions (06/21/04)	1	1	1	LVX TZP AMK IPM TIG
28 (B)	Environmental, laryngoscope (06/21/04)	1	1	1	LVX TZP AMK IPM RIF TIG
29 (B)	Central venous catheter (07/19/04)	1	1	1	LVX TZP AMK IPM RIF TIG
32 (B)	Respiratory secretions (07/13/04)	1	1	1	LVX TZP AMK IPM RIF TIG
50 (C)	Respiratory secretions (01/07/04)	4	1a	1	LVX TZP AMK IPM RIF SAM CS
62 (C)	Wound swab (03/19/04)	2	2	2	LVX TZP RIF TIG
63 (C)	Respiratory secretions (06/21/04)	1	1	1	LVX TZP AMK IPM SAM TIG
71 (C)	Environmental, desk surface (07/26/04)	1	1	1	LVX TZP AMK IPM SAM TIG
73 (E)	Respiratory secretions (05/28/05)	1	1	1	LVX TZP AMK IPM RIF
75 (C)	Wound swab (05/17/05)	2	2a	2	LVX TZP AMK RIF TIG
80 (D)	Wound swab (01/24/05)	1	1	1	LVX TZP AMK IPM RIF SAM TIG
82 (D)	Wound swab (04/12/04)	2	2	2	LVX TZP RIF TIG
86 (D)	Urine (04/11/04)	1	1	1	LVX TZP AMK IPM RIF TIG
87 (D)	Central venous catheter (07/06/04)	2	2	2	LVX TZP AMK TIG
88 (D)	Respiratory secretions (03/02/04)	2	2	2	LVX TZP RIF TIG
89 (D)	Respiratory secretions (02/11/05)	2	2	2	LVX TZP RIF TIG
93 (D)	Central venous catheter (10/04/04)	1	1	1	LVX TZP AMK IPM SAM TIG
100 (F)	Respiratory secretions (03/01/05)	1	1	1	LVX TZP AMK IPM RIF SAM TIG
105 (C)	Cerebrospinal fluid (06/27/05)	Variant	4	3	LVX TZP AMK RIF SAM
115 (G) ^c^	Blood culture (06/10/05)	1	1	1	LVX TZP AMK IPM RIF SAM
RUH 134^d^	Urine (1982)	2	1	1	SAM
RUH 875^d^	Urine (1984)	1	2	2	SAM RIF

^a ^Data are from refs [13,26].

^b ^Isolates showing an intermediate level of susceptibility were classified as resistant.

^c ^*A. baumannii *index strain, also called ACICU [27,28].

^d ^Representative of the European clonal lineages I (RUH 875) and II (RUH 134) [29].

### Antimicrobial agents and MIC assays

Antimicrobial agents were: levofloxacin, piperacillin-tazobactam, amikacin, imipenem, rifampicin, ampicillin-sulbactam, colistin, and tigecycline. MIC determinations for all antibiotics were performed by the broth microdilution method, according to the Clinical and Laboratory Standards Institute (CLSI) protocol [32]. All powders were obtained from the Sigma-Aldrich (Milan, Italy), except tigecycline (Wyeth-Ayerst, Collegeville, Pennsylvania, USA). MICs were determined in 96-well microtiter plates (Costar, Cambridge, Massachusetts, USA) containing freshly prepared Mueller-Hinton broth (Oxoid, Milan, Italy), to prevent oxidative degradation of tigecycline in aqueous solution (Wyeth Research, unpublished data). The inoculum was adjusted to ~5 × 10^5 ^CFU/ml in a 100-μl final volume, and microtiter plates were visually read after incubation for 24 h at 37°C. *Escherichia coli *ATCC 25922 and *Staphylococcus aureus *ATCC 29213 were used as internal quality control strains. The US FDA breakpoints approved for *Enterobacteriaceae *were applied to define tigecycline susceptibility (susceptibility, ≤ 2 mg/L; resistance, ≥ 8 mg/L). MIC results were interpreted according to the CLSI breakpoint criteria [32]. The criteria proposed by Gales et al were used for interpretation of colistin susceptibility [33]. Breakpoints for rifampicin were interpreted according to Hogg et al [34].

### Chequerboard assay

Antibiotic interactions were determined using the chequerboard assay as previously described [22]. The range of drug concentration used in the chequerboard analysis was such that the dilution range encompassed the MIC for each drug used in the analysis. Broth microdilution plates were inoculated with each *A. baumannii *isolate to yield ~5 × 10^5 ^CFU/ml in a 100-μl final volume, and incubated for 18 h at 37°C.

Synergy has been defined as requiring a fourfold reduction in the MIC of both antibiotics in combination, compared with each used alone, measuring the fractional inhibitory concentration index (FICI). The FICI was calculated for each combination using the following formula: FICI = FIC_A _+ FIC_B_, where FIC_A _= MIC of drug A in combination/MIC of drug A alone, and FIC_B _= MIC of drug B in combination/MIC of drug B alone. The FICI was interpreted as follows: synergy, FICI ≤ 0.5; indifference, 0.5 < FICI ≤ 4; antagonism, FICI > 4 [22].

### Time-kill assays

Tubes containing freshly prepared Mueller-Hinton broth supplemented with the drug were inoculated with *A. baumannii *isolates to a density of ~5 × 10^5 ^CFU/ml in a final volume of 10 ml and incubated in a shaking bath at 37°C. Aliquots were removed at time 0, 3, 6, and 24 h post-inoculation, and serially diluted in saline for determination of viable counts. Diluted samples (100 μl) were plated on Mueller Hinton agar plates and bacterial counts were determined after 18-h incubation at 37°C. The antibiotic concentrations used in time-kill assays corresponded to 0.5-, 1-, and 2-fold the MIC values in combination as determined by the chequerboard method, i.e. 2- to 16-fold lower than the MIC of each antibiotic alone (see Results). The bactericidal activity was defined as = 3 log_10 _CFU/ml reduction in the colony count relative to the initial inoculum [21]. Synergy was interpreted as ≥ 2 log_10 _decrease in CFU/ml by the drug combination when compared with its most active constituent, and = 2 log_10 _decrease in the CFU/ml below the initial inoculum, at any time point. The drug combination was considered to be antagonistic for = 2 log_10 _increase in CFU/ml and indifferent for < 2 log_10 _change in CFU/ml [22]. All synergistic interactions were confirmed by triplicate assays. Only antibiotic combinations showing synergism or antagonism in both chequerboard and time-kill assays were accepted as authentic synergistic or antagonistic interactions, respectively.

### Determination of mutation frequencies for resistance to antibiotics

This was performed essentially as described by Miller *et al*. [35]. Approximately 10^8 ^cells from overnight cultures in Mueller-Hinton broth were spread onto triplicate Mueller-Hinton agar plates supplemented with the selective antibiotic at a concentration that was four-fold higher than the respective MIC for an individual isolate. After 48 h incubation at 37°C, the number of colonies was counted, and mutation frequencies were expressed as the mean number of colonies recovered as a fraction of total viable bacteria plated. Isolates with mutation rates >10^-7 ^were considered to be mutators [36].

### Detection of ade genes for active efflux systems

The presence of *adeB*, *adeJ*, *adeE*, and *adeY*, and of the two-component regulatory system *adeRS *which controls AdeABC expression was investigated by PCR as previously reported [37-39]. The identity of *adeB*, *adeJ*, *adeR*, and *adeS *amplicons was confirmed by direct DNA sequencing.

## Results

The antibiotic susceptibility levels, expressed as MIC of levofloxacin, piperacillin-tazobactam, amikacin, imipenem, rifampicin, ampicillin-sulbactam, colistin and tigecycline, were preliminarily determined for the whole panel of 24 *A. baumannii *isolates [see Additional file 1]. All isolates, except the prototypic strains for the European clonal lineages I (RUH 875) and II (RUH 134), were resistant to levofloxacin and piperacillin-tazobactam (22 isolates each, 91.7%). A high percentage of isolates were resistant to amikacin (18 isolates, 75.0%), imipenem (15 isolates, 62.5%), and rifampicin (18 isolates, 75.0%), while only 9 (37.5%) and 1 (4.2%) isolates were resistant to ampicillin-sulbactam and colistin, respectively. Eighteen isolates (75.0%) were non-susceptible to tigecycline, including both resistant and intermediate phenotypes. Reference strains RUH 875 and RUH 134, isolated in early 1980s, showed an overall susceptible profile (Tables 1 and Additional file 1).

Chequerboard analysis performed with all antimicrobials in combination with tigecycline showed 5.9% synergy, 85.7% indifference, and 8.3% antagonism (Table 2). Tigecycline exerted synergistic activity with levofloxacin (4 isolates), amikacin, imipenem and colistin (2 isolates each). Notably, synergistic effects were observed only among tigecycline non-susceptible isolates (Table 2). Antagonistic interactions were frequently observed for tigecycline/piperacillin-tazobactam (8 isolates), and to a lesser extent for tigecycline/amikacin (3 isolates), tigecycline/colistin, tigecycline/ampicillin-sulbactam and tigecycline/rifampicin (1 isolate each) (Table 2). The concentration of individual drugs in synergistic combinations is shown in Table 3.

**Table 2:** Chequerboard results obtained with tigecycline in combination with seven antibiotics in 24 *A. baumannii *isolates

	Effect (FICI value) of TIG in combination with ^a^						
	
Study code	LVX	TZP	AMK	IPM	RIF	SAM	CS
5	**Sy (0.31)**	In (2.03)	*An (4.06)*	In (0.75)	In (0.62)	In (1.25)	In (0.62)
11	**Sy (0.31)**	In (2.03)	**Sy (0.50)**	In (0.75)	In (0.62)	In (1.50)	In (0.56)
16	**Sy (0.50)**	In (1.03)	In (0.62)	In (0.75)	In (1.06)	In (1.50)	**Sy (0.50)**
28	In (0.62)	In (2.03)	*An (8.06)*	In (0.75)	In (1.00)	In (1.50)	In (0.56)
29	In (0.75)	In (2.06)	In (0.75)	In (0.75)	In (1.00)	In (1.50)	In (1.12)
32	In (1.12)	In (2.06)	In (1.25)	In (0.75)	In (1.00)	In (1.50)	In (0.62)
50	In (0.75)	*An (8.03)*	In (0.75)	In (0.75)	In (0.75)	In (2.50)	In (0.56)^b^
62	In (1.00)	In (1.00)	In (1.00)	**Sy (0.37)**	In (0.62)	In (1.00)	In (0.62)
63	In (0.56)	In (2.03)	In (0.56)	In (0.75)	In (0.75)	In (1.25)	In (0.56)
71	In (0.75)	In (2.03)	**Sy (0.50)**	In (0.75)	In (0.56)	In (1.25)	In (0.56)
73	In (0.75)	*An (4.06)*	In (1.12)	In (1.12)	In (1.50)	In (2.50)	*An (4.25)*
75	**Sy (0.31)**	In (0.75)	In (0.62)	In (0.62)	In (0.62)	In (0.56)	**Sy (0.50)**
80	In (0.62)	*An (4.06)*	In (0.62)	**Sy (0.37)**	In (1.00)	In (1.25)	In (0.62)
82	In (0.56)	In (0.75)	In (2.12)	In (1.25)	In (0.62)	In (0.75)	In (1.25)
86	In (1.00)	*An (4.06)*	In (1.00)	In (0.75)	In (1.00)	In (1.50)	In (0.56)
87	In (0.56)	In (0.75)	In (0.56)	In (0.75)	In (2.12)	In (0.75)	In (1.12)
88	In (0.62)	In (1.00)	*An (4.03)*	In (0.75)	In (0.75)	In (0.62)	In (0.75)
89	In (0.62)	In (1.50)	In (1.06)	In (0.75)	In (0.75)	In (0.75)	In (1.12)
93	In (0.56)	*An (4.06)*	In (2.06)	In (1.00)	*An (4.25)*	In (1.00)	In (0.62)
100	In (0.56)	In (2.03)	In (1.00)	In (1.00)	In (1.00)	In (0.75)	In (0.62)
105	In (2.03)	*An (4.03)*	In (0.75)	In (2.50)	In (2.50)	*An (4.25)*	In (2.25)
115	In (1.06)	*An (4.06)*	In (2.25)	In (1.50)	In (1.50)	In (2.25)	In (1.06)
RUH 134	In (1.50)^c^	*An (4.12)*	In (0.75)	In (1.50)^c^	In (1.00)	In (2.12)	In (1.06)
RUH 875	In (1.50)^c^	In (2.06)	In (2.12)	In (1.00)^c^	In (1.00)	In (2.06)	In (2.12)

^a ^Synergistic and antagonistic interactions are in bold and italics, respectively.

^b ^The FICI value of the tigecycline/colistin combination has been calculated considering a MIC value of 32 mg/L for colistin [see Additional file 1].

^c ^FICI value of tigecycline in combination with levofloxacin and imipenem has been calculated considering a MIC value of 0.125 mg/L for both levofloxacin and imipenem [see Additional file 1].

**Table 3:** MIC values for individual antibiotics alone (as determined by the broth microdilution method) and in effective synergistic combination with tigecycline (as determined by the chequerboard method) for seven *A. baumannii *isolates

	MIC (mg/L) ^a^								
	
Study code	TIG	LVX	AMK	IPM	CS	TIG/LVX	TIG/AMK	TIG/IPM	TIG/CS
5	4	16	64	16	0.25	0.25/4	NS	NS	NS
11	4	16	64	16	0.5	0.25/4	1/16	NS	NS
16	8	16	128	16	0.125	2/4	NS	NS	2/0.03
62	4	8	2	2	0.25	NS	NS	0.25/0.5	NS
71	4	8	256	16	0.5	NS	1/64	NS	NS
75	4	16	128	2	0.5	0.25/4	NS	NS	1/0.125
80	4	8	128	32	0.25	NS	NS	0.25/8	NS

^a ^Breakpoint criteria are provided in Additional file 1.

**Figure 1:**
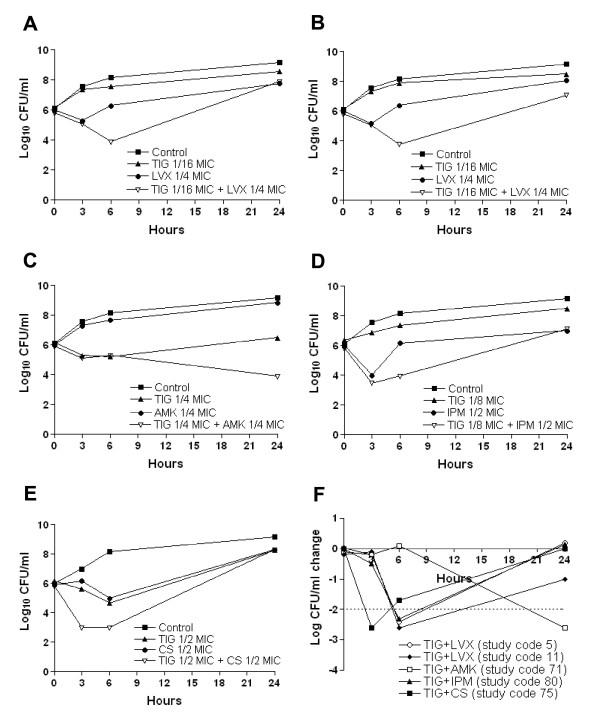
**Time-kill kinetics for confirmed synergistic interactions**. (A) TIG/LVX, study code 5; (B) TIG/LVX, study code 11; (C) TIG/AMK, study code 71, (D) TIG/IPM, study code 80; (E) TIG/CS, study code 75; (F) Comparison of quantitative change in CFU/ml, relative to the most active constituent, for the synergistic interactions. The drug concentrations are as follows: TIG/LVX, 0.25 and 4 mg/L, respectively (study codes 5 and 11); TIG/AMK, 1 and 64 mg/L, respectively (study code 71); TIG/IPM, 0.5 and 16 mg/L, respectively (study code 80); TIG/CS, 2 and 0.25 mg/L, respectively (study code 75). The dotted line denotes the threshold value to define synergy. Panels show one representative experiment of three replicates.

All synergistic interactions inferred from chequerboard analysis were reassessed by time-kill kinetic experiments performed with tigecycline in combination with levofloxacin, amikacin, imipenem and colistin. Time-kill diagrams for effective combinations are shown in Figure 1. Five of 10 synergistic combinations, namely tigecycline/levofloxacin (2 out of 4 isolates), tigecycline/amikacin, tigecycline/imipenem and tigecycline/colistin (1 out of 2 isolates each) were confirmed. Synergistic effects were observed at 3 h for tigecycline/colistin (2/0.25 mg/L = 1/2 MIC for both antibiotics), at 6 h for tigecycline/imipenem (0.5/16 mg/L = 1/2 MIC for tigecycline and 1/8 MIC for imipenem) and tigecycline/levofloxacin (0.25/4 mg/L = 1/16 MIC for tigecycline and 1/4 MIC for levofloxacin), and at 24 h for tigecycline/amikacin (1/64 mg/L = 1/4 MIC for both antibiotics). Re-growth was observed after 24 h for tigecycline/colistin, tigecycline/imipenem and tigecycline/levofloxacin combinations (Figure 1). No synergistic combination resulted in bactericidal activity. Moreover, none of the 14 antagonistic interactions inferred from chequerboard analysis was confirmed by time-kill assays (data not shown).

To determine if regrowth in time-kill assays was due to *A. baumannii *hypermutability, the spontaneous mutation frequency toward antibiotic resistance was determined for isolates showing regrowth at 24 h (study codes 5, 11, 75, 80) or not (study code 71). Resistant mutants were not detected (mutation rate <10^-8^) upon selection with levofloxacin, amikacin, imipenem, and colistin at four-fold the MIC, while tigecycline-resistant mutants generated with a frequency between 1.2 × 10^-8 ^and 9.0 × 10^-8 ^for all tested isolates (see Additional file 2).

Search for efflux genes showed that all isolates were positive for the *adeB *and *adeJ *genes and negative for both *adeE *and *adeY *genes, irrespective of their resistance or susceptibility profile. All of the isolates were positive for the two-component regulatory system *adeRS*.

## Discussion

In this work we investigated tigecycline interactions with various antimicrobials by a two-step approach, involving preliminary chequerboard screening and subsequent time-kill assays. The chequerboard is an easy to perform, high-throughput method which provides single time-point evidence of bacterial growth inhibition, and generally results in an overestimate of synergistic interactions [22]. For these reasons, all effective combinations inferred from chequerboard analysis were reassessed by time-kill assays. Although time consuming and cumbersome, the time-kill assays provide a dynamic picture of antibiotic action over time [40]. Hence, only combinations showing synergy in both assays were interpreted as authentic synergistic interactions.

While chequerboard screening provided synergistic results for the combinations tigecycline/levofloxacin, tigecycline/amikacin, tigecycline/imipenem and tigecycline/colistin in 7 out of 24 isolates, time-kill kinetics confirmed synergism in only 5 out of 7 isolates, 4 of which were resistant to carbapenems and belonged to type 1 (related to the European clonal lineage II) [26]. The different synergistic activities observed in *A. baumannii *isolates sharing the same epidemiological type [26] probably reflect the variable expression of different resistance determinants. This poses the need to test synergistic interactions even in case of clonal isolates characterized by identical genetic fingerprint and resistance profile.

Three out of five tigecycline synergistic concentrations observed in this study with time-kill assays exceed the maximum plasmatic concentration of tigecycline (0.38 mg/L) achievable with a standard dosage [41]. However, thanks to its pharmacodynamic properties, tigecycline is rapidly distributed into tissues resulting in up to 78-fold higher tissue concentrations, compared to plasma [42,43]. These considerations suggest a clinical usefulness for some of the synergistic combinations here detected for tigecycline.

We identified one isolate (study code 71) showing tigecycline/amikacin synergistic interaction at 24 h (1 and 64 mg/L for tigecycline and amikacin, respectively). This strain was resistant to amikacin (MIC = 256 mg/L) and showed an intermediate resistance to tigecycline (MIC = 4 mg/L). Although the synergistic concentration for amikacin (64 mg/L) is significantly above the threshold achievable in clinical treatments with a multi daily dosing regimen (20–30 mg/L), higher concentrations (65–75 mg/L) can be achieved with a single amikacin daily dose [44].

*In vitro *synergistic interactions between tigecycline and colistin have previously been demonstrated by time-kill assays in *Klebsiella pneumoniae *[45] and *in vivo *for the treatment of a severe case of MDR *Pseudomonas aeruginosa *osteomyelitis [46]. Although several studies have reported clinical efficacy of colistin [7,8], the synergistic effect of tigecycline/colistin combination has never been demonstrated in *A. baumannii *by time-kill analysis. Here, we showed for one *A. baumannii *isolate (study code 75) a synergistic effect at 3 h of incubation for tigecycline/colistin combination (2 and 0.25 mg/L for tigecycline and colistin, respectively), with a subsequent re-growth within 24 h. This strain was susceptible to colistin (MIC = 0.5 mg/L) and intermediate resistant to tigecycline (MIC = 4 mg/L). Notably, the colistin synergistic concentration is significantly below the serum concentration achievable after standard dosing regimen (5–6 mg/L) [44,47]. As noted by various authors [48-50], colistin causes permeabilisation of the bacterial outer membrane, which would allow enhanced penetration by and activity of the other antibiotic in combination. The tigecycline/colistin synergistic interaction could therefore have an impact in clinical practice by reducing the therapeutic dosage of colistin, and hence the risk of collateral effects which currently represent a major limitation to its clinical use [7,8].

We also demonstrated a synergistic interaction for the combination tigecycline/imipenem (0.5 and 16 mg/L for tigecycline and imipenem, respectively) in one *A. baumannii *isolate (study code 80), belonging to the epidemic type 1, and carrying the *bla*_OXA-58 _gene [26]. It is of note that the serum concentration achievable during imipenem treatment is 20 mg/L [47]. Thus, the synergistic interaction tigecycline/imipenem, which has never been described before for *A. baumannii*, could represent a valid therapeutic option to combat the increasingly frequent *A. baumannii *isolates resistant to both these drugs.

Resistance to quinolones is widespread among MDR *A. baumannii *strains [51]. In this study, a high percentage of *A. baumannii *isolates were resistant to levofloxacin as a single agent. Here we report for the first time a synergistic interaction between tigecycline and levofloxacin (0.25 and 4 mg/L respectively) for 2 *A. baumannii *isolates, at 6 h of incubation. These strains showed full resistance to levofloxacin (MIC = 16 mg/L) and intermediate resistance to tigecycline (MIC = 4 mg/L). Also in this instance, the levofloxacin synergistic concentration is below the maximum serum concentration (5.9 mg/L) [52].

Even if no undesirable antagonistic combinations were confirmed in this study by time-kill assay, we detected a decreased antimicrobial efficacy for the tigecycline/piperacillin-tazobactam combination, compared to the antimicrobial efficacy of piperacillin-tazobactam alone (data not shown). This result is worrying considering that tigecycline/piperacillin-tazobactam combination therapy is often given empirically, without the support of *in vitro *interaction assays.

The molecular mechanisms of synergy between tigecycline and the various antibiotics deserve further investigation. Overexpression of the AdeABC efflux pump has been demonstrated in tigecycline resistant *A. baumannii *isolates [53], and our results indicate that all *A. baumannii *isolates tested carry the *adeABC/adeIJK *genes, suggesting that their variable expression level – but not their presence *per se *– could contribute to the extent of resistance. We also showed that *adeDE *is not present in *A. baumannii*, in agreement with previous studies [38,39].

The regrowth after 24 h observed in time-kill experiments for all confirmed synergistic combination, except for tigecycline/amikacin, could reflect the labile nature in solution of tigecycline due to oxidative degradation (Wyeth Research, unpublished data) and/or the tendency of *A. baumannii *strains to induce resistance on exposure to antimicrobial agents, especially at sub-MIC concentrations. At present, we are unable to check the tigecycline levels and therefore we cannot determine if tigecycline was degraded, at least partially, during the experimental time course.

Determination of mutation frequencies for resistance to levofloxacin, amikacin, imipenem, colistin, and tigecycline at four-fold the MIC failed to detect any hypermutator phenotype for all isolates showing synergy in time-kill assays, irrespective of regrowth. Moreover, the mutation frequency toward resistance to tigecycline (~5 × 10^-8^) or other antibiotics (<10^-8^) is incompatible with the observed regrowth kinetics (Figure 1). Hence, we can only speculate that regrowth was due to different response of isolates to antibiotic-induced overexpression of broad-specificity multidrug efflux systems, like AdeABC and AdeIJK [16,37-39], rather than hypermutability. According to this hypothesis, the tigecycline/amikacin interaction may have prevented the expression of efflux-based resistance by a still undefined mechanism, ultimately resulting in more effective synergism. In fact, a recent study on tigecycline/amikacin synergistic interactions in *A. baumannii *demonstrated the suppression of regrowth at 24 h for this particular antibiotic combination, in full agreement with our findings [25].

Further studies are needed to elucidate the molecular mechanisms responsible for synergistic interactions with tigecycline and to explore their therapeutic potential. It will also be necessary to combine *in vitro *findings with additional pharmacokinetic and pharmacodynamic data in order to provide more meaningful prediction of the *in vivo *efficacy of synergistic combinations in clinical practice. Lastly, *in vitro *synergy testing of tigecycline combinations is recommended prior to starting any combined therapy for treatment of infections sustained by MDR and pan-resistant *A. baumannii*.

## Abbreviations

AMK: amikacin; CS: colistin; IPM: imipenem; LVX: levofloxacin; RIF: rifampicin; SAM: ampicillin-sulbactam; TIG: tigecycline; TZP: piperacillin-tazobactam; Sy: synergy; In: indifference; An: antagonism; NS: not synergic; S: susceptible; I: intermediate; R: resistant.

## Competing interests

The authors declare that they have no competing interests.

## Authors' contributions

LP and SD performed the susceptibility tests, the molecular genetics studies and drafted part of the manuscript. AC participated in the design of the study, provided clinical interpretation of susceptibility data, and drafted part of the manuscript. NP and PV conceived of the study, participated in its design and coordination, and critically revised the draft manuscript. All authors read and approved the final manuscript.

## Supplementary Material

Additional file 1 **Distribution of MIC values and antibiotic susceptibility profile for 24 *A. baumannii *isolates**. The data provided are MIC values and antibiotic susceptibility categories (S, I, R) for all strains analysed in this study. Click here for file

Additional file 2 **Mutation frequency for resistance to antibiotics at four-fold the MIC**. The data provided are mutation frequencies for resistance to TIG, LVX, AMK, IPM and CS for strains showing authentic synergism according to the definition provided in Methods. Click here for file
