# Supplementary material for: In vitro activity of tigecycline in combination with various antimicrobials against multidrug resistant Acinetobacter baumannii
Source: Ann Clin Microbiol Antimicrob. 2009 May 21;8:18. doi: 10.1186/1476-0711-8-18 (PMC2693502; doi:10.1186/1476-0711-8-18)
Supplement: Additional file 1 — Distribution of MIC values and antibiotic susceptibility profile for 24 A. baumannii isolates. The data provided are MIC values and antibiotic susceptibility categories (S, I, R) for all strains analysed in this study. [file 1476-0711-8-18-S1.doc]

**Additional file 1**

**Distribution of MIC values and antibiotic susceptibility profile for 24 *A. baumannii* isolates.**

| Study code | MIC (mg/L) and susceptibility ^a^ | | | | | | | |
| --- | --- | --- | --- | --- | --- | --- | --- | --- |
|  | TIG | LVX | TZP | AMK | IPM | RIF | SAM | CS |
| 5 | 4 (I) | 16 (R) | 1024 (R) | 64 (R) | 16 (R) | 2 (R) | 16/8 (I) | 0.25 (S) |
| 11 | 4 (I) | 16 (R) | 1024 (R) | 64 (R) | 16 (R) | 2 (R) | 8/4 (S) | 0.5 (S) |
| 16 | 8 (R) | 16 (R) | 1024 (R) | 128(R) | 16 (R) | 1 (S) | 8/4 (S) | 0.125 (S) |
| 28 | 4 (I) | 8 (R) | 1024 (R) | 64 (R) | 16 (R) | 2 (R) | 8/4 (S) | 0.5 (S) |
| 29 | 4 (I) | 8 (R) | 512 (R) | 64 (R) | 16 (R) | 2 (R) | 8/4 (S) | 0.25 (S) |
| 32 | 4 (I) | 8 (R) | 512 (R) | 32 (I) | 16 (R) | 2 (R) | 8/4 (S) | 0.25 (S) |
| 50 | 1 (S) | 16 (R) | 1024 (R) | 128(R) | 128 (R) | 32 (R) | 32/16 (R) | >32 (R) |
| 62 | 4 (I) | 8 (R) | 64 (I) | 2 (S) | 2 (S) | 8 (R) | 4/2 (S) | 0.25 (S) |
| 63 | 4 (I) | 8 (R) | 1024 (R) | 256 (R) | 16 (R) | 0.25 (S) | 16/8 (I) | 0.5 (S) |
| 71 | 4 (I) | 8 (R) | 1024 (R) | 256 (R) | 16 (R) | 1 (S) | 16/8 (I) | 0.5 (S) |
| 73 | 2 (S) | 8 (R) | 512 (R) | 128 (R) | 32 (R) | 2 (R) | 8/4 (S) | 0.25 (S) |
| 75 | 4 (I) | 16 (R) | 128 (R) | 128 (R) | 2 (S) | 2 (R) | 8/4 (S) | 0.5 (S) |
| 80 | 4 (I) | 8 (R) | 256 (R) | 128 (R) | 32 (R) | 2 (R) | 16/8 (I) | 0.25 (S) |
| 82 | 4 (I) | 64 (R) | 128 (R) | 8 (S) | 1 (S) | 2 (R) | 8/4 (S) | 0.125 (S) |
| 86 | 4 (I) | 8 (R) | 256 (R) | 32 (I) | 16 (R) | 2 (R) | 8/4 (S) | 0.25 (S) |
| 87 | 4 (I) | 64 (R) | 256 (R) | 32 (I) | 1 (S) | 1 (S) | 8/4 (S) | 0.25 (S) |
| 88 | 8 (R) | 64 (R) | 64 (I) | 8 (S) | 1 (S) | 2 (R) | 8/4 (S) | 0.5 (S) |
| 89 | 8 (R) | 64 (R) | 64 (I) | 16 (S) | 1 (S) | 2 (R) | 8/4 (S) | 0.25 (S) |
| 93 | 4 (I) | 32 (R) | 256 (R) | 256 (R) | 8 (I) | 0.5 (S) | 16/8 (I) | 0.25 (S) |
| 100 | 4 (I) | 8 (R) | 1024 (R) | 32 (I) | 32 (R) | 2 (R) | 16/8(I) | 0.25 (S) |
| 105 | 0.5 (S) | 64 (R) | 1024 (R) | 128 (R) | 4 (S) | 2 (R) | 16/8 (I) | 0.125 (S) |
| 115 | 2 (S) | 4 (I) | 512 (R) | 64 (R) | 8 (I) | 2 (R) | 16/8 (I) | 0.5 (S) |
| RUH 134 | 2 (S) | <0.125 (S) | 4 (S) | 4 (S) | <0.125 (S) | 2 (R) | 32/16 (R) | 0.5 (S) |
| RUH 875 | 1 (S) | <0.125 (S) | 8 (S) | 8 (S) | <0.125 (S) | 1 (S) | 64/32 (R) | 0.25 (S) |

^a^ Breakpoint criteria were as follows. TIG: susceptible, ≤ 2 mg/L; intermediate, 4 mg/L; resistant, ≥ 8 mg/L according to FDA; LVX: susceptible, ≤ 2 mg/L; intermediate, 4 mg/L; resistant, ≥ 8 mg/L [32]; TZP: susceptible, ≤ 16/4 mg/L; intermediate, 32/4-64/4 mg/L; resistant, ≥ 128/4 mg/L [32]; AMK: susceptible, ≤ 16 mg/L; intermediate, 32 mg/L; resistant, ≥ 64 mg/L [32]; IPM: susceptible, ≤ 4 mg/L; intermediate, 8 mg/L; resistant, ≥ 16 mg/L [32]; RIF: susceptible, ≤ 1 mg/L; resistant, ≥ 2 mg/L [34]; SAM: susceptible, ≤ 8/4 mg/L; intermediate, 16/8 mg/L; resistant, ≥ 32/16 mg/L [32]; CS: susceptible, ≤ 2 mg/L; resistant ≥ 4 mg/L [33].
