# Supplementary material for: In vitro activity of tigecycline in combination with various antimicrobials against multidrug resistant Acinetobacter baumannii
Source: Ann Clin Microbiol Antimicrob. 2009 May 21;8:18. doi: 10.1186/1476-0711-8-18 (PMC2693502; doi:10.1186/1476-0711-8-18)
Supplement: Additional file 2 — Mutation frequency for resistance to antibiotics at four-fold the MIC. The data provided are mutation frequencies for resistance to TIG, LVX, AMK, IPM and CS for strains showing authentic synergism according to the definition provided in Methods. [file 1476-0711-8-18-S2.doc]

**Additional file 2**

**Mutation frequency for resistance to antibiotics at four-fold the MIC.**

| Study code | Mutation frequency for resistance to antibiotic (mg/L)^a^ | | | | |
| --- | --- | --- | --- | --- | --- |
|  | TIG | LVX | AMK | IPM | CS |
| 5 | 5.7×10^-8^ (16) | <10^-8^ (64) | <10^-8^ (256) | <10^-8^ (64 ) | <10^-8^ (1) |
| 11 | 2.3×10^-8^ (16) | <10^-8^ (64) | <10^-8^ (256) | <10^-8^ (64) | <10^-8^ (2) |
| 71 | 4.4×10^-8^ (16) | <10^-8^ (32) | <10^-8^ (1024) | <10^-8^ (64) | <10^-8^ (2) |
| 75 | 1.2×10^-8^ (16) | <10^-8^ (64) | <10^-8^ (512) | <10^-8^ (8) | <10^-8^ (2) |
| 80 | 9.0×10^-8^ (16) | <10^-8^ (32) | <10^-8^ (512) | <10^-8^ (128) | <10^-8^ (1) |

^a^ The antibiotic concentrations (in brackets) are 4×MIC determined for individual isolates.
